# Comparative Bioavailability of Two Diosmin Formulations after Oral Administration to Healthy Volunteers

**DOI:** 10.3390/molecules23092174

**Published:** 2018-08-29

**Authors:** Rosario Russo, Divya Chandradhara, Nunziatina De Tommasi

**Affiliations:** 1 Giellepi S.p.A. Health Science, via B. Cellini 37, 20851 Lissone (MB), Italy; 2 Bioagile Therapeutics Pvt. Ltd., Bangalore 560094, India; divya@bioagiletherapeutics.com; 3 Dipartimento di Farmacia, Università di Salerno, via Giovanni Paolo II 132, 84084 Fisciano (SA), Italy; detommasi@unisa.it

**Keywords:** µSmin^®^ Plus, diosmin, diosmetin, pharmacokinetics, chronic venous insufficiency (CVI)

## Abstract

Diosmin is a flavonoid commonly found in citrus fruits, largely used as adjuvant treatment for circulatory disorders, including chronic venous insufficiency (CVI) and hemorrhoids. Following oral administration, diosmin is not directly absorbed but must first be hydrolyzed into its aglycone, diosmetin, which is then absorbed into the systemic circulation. The aim of the current cross-over clinical study was to assess the pharmacokinetic profile of µSmin^®^ Plus, a micronized diosmin flavonoid complex standardized in diosmin and formulated with a buffering agent (tested formulation). The study compared this to unformulated micronized diosmin (reference), in 16 healthy volunteers. Plasma samples were analyzed by HPLC-MS and plasma diosmetin concentration was measured after deconjugation with β-glucuronidase. For the tested formulation area under the curve (AUC_0-t_), and maximum plasma and time concentration (C_max_; t_max_) were found to be 298.4 ± 163.7, 50.3 ± 22.6 and 2.2 ± 2.9, respectively. AUC_0-t_ and C_max_ of the reference were 31.9 ± 100.4 and 2.4 ± 1.9, respectively. The tested formulation showed higher plasmatic concentrations of diosmetin in comparison to those obtained after the administration of unformulated micronized diosmin. The relative bioavailability was 9.4 greater for the tested formulation than in micronized diosmin. In conclusion, our data indicate that µSmin^®^ Plus was rapidly and well absorbed into systemic circulation and may therefore be ideally suitable to deliver diosmin in human interventional trials.

## 1. Introduction

**Figure 1:**
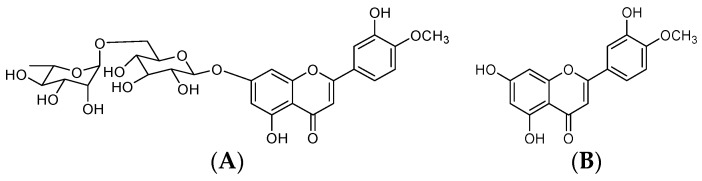
Chemical structure of diosmin (**A**) and diosmetin (**B**).

Diosmin, 3′,5,7-trihydroxy-4′-methoxyflavone-7-rutinoside (Figure 1), is a flavonoid commonly present in most plants and fruits, mainly those belonging to *Citrus* spp. [1,2,3]. In Europe, diosmin has been widely used for a long time as a phlebotonic and vascular protector for oral use in different types of pharmaceutical and nutritional products. Thanks to its biological activity and safety profile, diosmin is considered to be a valid therapeutic tool for the management of chronic venous insufficiency (CVI), hemorrhoids, lymphedema, and varicose veins [4]. Nevertheless, most flavonoids, including diosmin, are poorly soluble, leading to a low dissolution rate and impaired uptake from the gastrointestinal tract [5]. Following oral administration, diosmin is quickly hydrolyzed into its aglycone, diosmetin, by enzymes from intestinal microflora. Resulting diosmetin is then absorbed through the intestinal wall. When in systemic circulation, it is enzymatically esterified to its most relevant metabolite, the 3-*O*-glucuronide, which is further esterified to 3, 7-*O*-diglucuronide [6,7]. However, the amount of diosmetin detected in plasma after a single oral administration of diosmin is low, highly variable, and often inconsistent across different studies. According to published literature, plasma concentrations are marginally ameliorated when diosmin is administered as a micronized form, due to the particle size reduction effect on intestinal absorption [5]. Although the micronization process can be considered a consolidated technology to improve bioavailability of poorly soluble substances, further increases in oral bioavailability are required to achieve optimal therapeutic efficacy.

The aim of the current double-blind, two-period, cross-over clinical study in healthy volunteers was to: compare the measurement of diosmetin (a diosmin metabolite) in plasma after the oral administration of a single dose of µSmin^®^ Plus (a diosmin flavonoid complex) (tested formulation), against unformulated micronized diosmin (reference).

## 2. Results

Sixteen healthy male subjects (mean age: 29.9 ± 6.1 years; mean weight: 63.7 ± 7.0 kg; mean height: 166.0 ± 6.0 cm; body mass index: 23.0 ± 1.8; Table 1) participated in the current clinical trial and all completed the study. Neither the tested formulation, nor the reference, changed the basic physiological parameters (blood pressure, heart rate, respiratory rate and body temperature).

**Figure 2:**
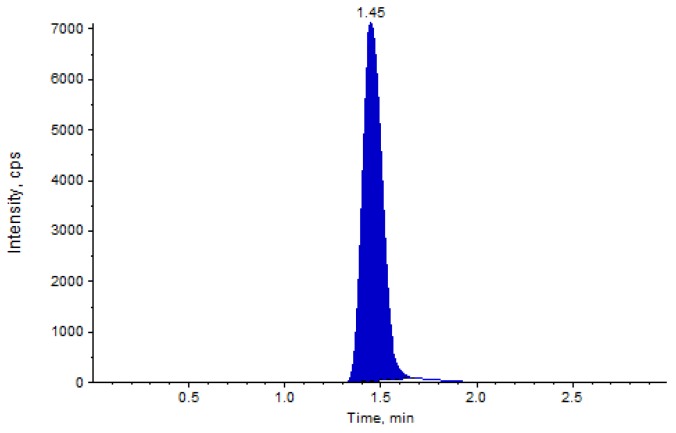
LC-MS/MS chromatogram of diosmetin from plasma sample of a treated volunteer.

HPLC-MS analysis showed good separation of analytes, namely the diosmetin and internal standard. The linear calibration curves in the examined concentration range of 0.506 to 211.863 ng/mL showed a R^2^ ≥ 0.996, and precision and accuracy values within the tolerated limits of operating guidelines (Table 2). An example of LC-MS/MS chromatogram is shown in Figure 2. 

**Figure 3:**
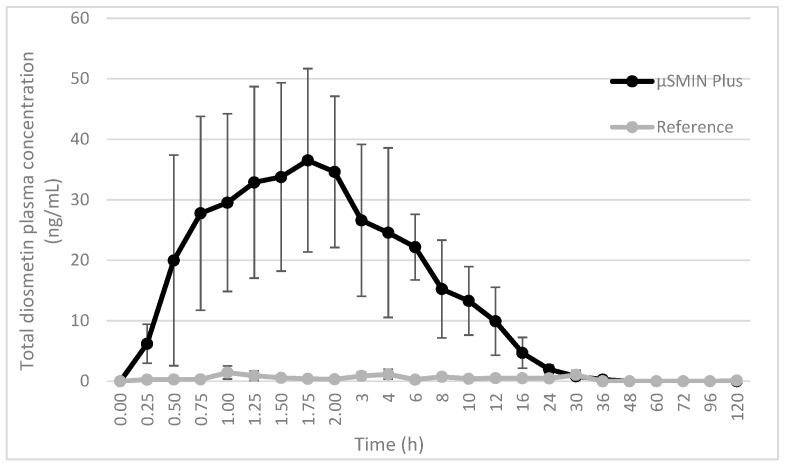
Plasma concentration time curves of diosmetin after a single oral administration of a tablet containing either µSMIN^®^ Plus or micronized diosmin, in healthy male subjects. Each point represents the mean ± standard deviation (SD) of 16 volunteers.

Plasma diosmetin, the major metabolite of diosmin, was measured in 16 subjects to assess diosmin bioavailability. After dosing the tested formulation (T), plasma concentrations of diosmetin were higher than those found after dosing with the reference (R) product (*p* < 0.001), as shown in the plasma concentration-time curves (Figure 3). After T administration, plasma concentrations were measured in 20 plasma samples from 24 samples collected from each volunteer. In contrast, after treatment with R timed plasma, concentrations were often lower than the limit of quantification (LOQ). Bioavailability of diosmetin was higher after administration of T compared to that found after R intake (*p* < 0.001). Interestingly, the variability of plasma concentrations in each subject, expressed as coefficient of variation (CV%), was relatively lower for T than R.

Table 3 shows mean values ± SD of pharmacokinetic parameters assessed in the current study, including the area under the curve (AUC_0-t_), maximum plasma and time concentration (C_max_; t_max_). CV (%) were significantly lower for T than R, showing a higher uniformity in the data. Only for t_1/2_ was the CV very high, even in the T group. This was due to the relevant data dispersion in the last β-phase of the plasma concentration curve, which have poor, if not nil, significance for the half-life definition. Concentrations of diosmetin were below the LOQ in samples from subjects receiving R, differently from what was found after the administration of T. For this reason, it was not possible to establish the t_max_ for reference. Relative bioavailability was 9.4-fold higher for T compared to the unformulated R product.

## 3. Discussion

Flavonoids are a class of phenolic compounds commonly present in plants with widely recognized biochemical and pharmacological actions. Under natural conditions, the majority of flavonoids exist in the form of glycoside (i.e., linked with various sugar moieties) which is poorly absorbed by the human gut. In order to become bioactive in the human body, these compounds need to be hydrolyzed to their aglycone form by enzymes found in the intestinal flora [8]. In particular, α-glucosidase and β-glucosidase are the main enzymes involved in the metabolism and deglycosylation of flavonoids [9,10,11]. Cleavage of the glycoside represents the first and crucial step for the intestinal absorption of diosmin [12,13,14] enzymatically hydrolyzed into diosmetin and its sugar moiety, immediately after oral administration. It is generally agreed that the sugar moiety is the major determinant of the flavonoids intestinal absorption, resulting in several times higher bioavailability of the aglycone, compared to the glycoside form. Bredsdorff et al. [12] conducted a double-blind, randomized, crossover clinical trial in 16 healthy volunteers. The volunteers consumed orange juice with natural content of rutinoside flavonoid (narirutin), or orange juice previously treated with α-rhamnosidase. An improvement of bioavailability was indicated in the latter, due to the conversion of the parent compound to its aglycone. 

Diosmetin, resulting from the hydrolysis of ingested diosmin, is absorbed most likely by intestinal cells through passive diffusion. However, the hypothesis of active transport should not be completely excluded according to the in vitro results obtained by Serra et al. [15] in two different experimental models, i.e., Caco-2 cells and PAMPA (parallel artificial membrane permeability assay). These data agree with those of Garner et al. [7] who investigated the enteral absorption of diosmin, mixed with trace of ^14^C-diosmin measuring the total radioactivity in urine and feces of 12 healthy volunteers [14]. The amount of diosmin absorbed from the radioactivity found in the urine showed that 57.9% of ingested diosmin was absorbed.

Several factors affect the intestinal absorption of molecules, such as lipophilicity, ionization degree and molecular size. It has been shown that dissociation of alcoholic groups found in diosmin molecule occurs at alkaline pH conditions. In particular, Serra et al. [15] showed an inflexion point in the ionization curves of diosmin, corresponding to an ionization constant (pK_a_) of 10. For the aglycone (diosmetin), the pK_a_ was 6 and 8 resulting in a better permeation across the cell membrane. No transport across the Caco-2 cell layer was detected for glycosylated flavonoid (diosmin). These results agree with data published by Fraeg et al. [16] showing that diosmin is completely dissociated at high pH value. Moreover, it appears that in such conditions, the crude diosmin did not pass the intestinal barrier in an ex vivo model of a non-everted rat gut sac. Whilst a diosmin nanosuspension formulated with stabilizers showed good permeability, likely due to the nano-dimension of diosmin. Unless in the presence of high pH, solubility of diosmin is poor. According to the tested formulation results, it is highly likely that the buffering agent used in µSmin^®^ Plus might exert the double action of sustaining the enzymatic reaction, which occurs naturally in the gut at slightly alkaline conditions, and at the same time preventing a dramatic increase in the intestinal pH, inducing the ionization of diosmetin [15].

Literature suggests that diosmin is a compound scarcely absorbed after oral administration. This affects the consistency of pharmacokinetic parameters, including its bioavailability, confirmed in the current reference product study, containing unformulated micronized diosmin. In order to achieve optimal absorption, the pre-systemic enzymatic hydrolysis releasing diosmetin and the systemic esterification of diosmetin, should be kept constant. Whereas the rate of enteral absorption of the aglycone should be increased by a promotor of epithelial permeability. Our hypothesis to ameliorate the enteral absorption of diosmin metabolic moiety was to allow an increase of diosmin biotrasformation to diosmetin in the gut, causing more relevant plasmatic concentrations in the systemic circulation. The fact that the administration of the formulated diosmin resulted in a significantly shorter time to peak plasma concentration, compared to standard diosmin, supports our hypothesis. Perhaps, this could also be explained by the dissolution efficiency of the formulated diosmin due to the granulation process of using the fluid bed. This is consistent with results of Takahashi et al. [17] indicating the dissolution rate of nimodipine and spironolactone tablets improved after bed fluid granulation.

The present crossover clinical study in healthy volunteers showed at least 9-fold higher systemic absorption over the reference product. Differences in C_max_ and AUC_0-t_ between formulated diosmin and unformulated diosmin were statistically significant. These agree with our previously published [18] results that confirmed a higher relative bioavailability of µSmin^®^ Plus vs. micronized diosmin (i.e., 4-fold), after oral administration of formulated diosmin in rats. 

Limited investigation over the years has been done into the improvement of the bioavailability of diosmin. Among them, the micronization technique has been found to be the most effective way to increase the solubility/bioavailability of poorly soluble compounds, such as flavonoids. However, the outcomes of this study indicate that different methods, such as those that favor the bioconversion of diosmin to its aglycone form, are a better way to achieve higher plasma concentration of diosmetin. These potentially increase the clinically efficacy of oral diosmin.

Further studies are needed to clearly elucidate the mechanism responsible for the enhanced intestinal absorption and improved bioavailability of µSmin^®^ Plus, and to determine its therapeutic dosage in humans.

## 4. Materials and Methods

### 4.1. Ethics

The Research Ethics Committee (Athiyandhal, Tiruvannamalai, India) approved the study protocol on 23rd June 2017. All enrolled subjects were able to comply with the study procedures and gave their written informed consent prior to initiation of the trial. Throughout the study, the principles of Declaration of Helsinki and further amendments were fully respected.

### 4.2. Study Design and Population

The current study was designed as a double-blind, two-period, cross-over clinical trial. A wash-out period of 2 weeks between two consecutive treatments was placed. The primary objective was to compare the pharmacokinetic profile of two oral tablets containing a formulated diosmin complex (T) and unformulated micronized diosmin (R), through plasma concentration of diosmetin, as the main metabolic by-product of ingested diosmin, in healthy volunteers. The secondary objective was to assess the tolerance and safety of the investigational products.

Sixteen healthy male volunteers were recruited for this study (age 29.9 ± 6.1 years; height 166.0 ± 6.0 cm; weight 63.7 ± 7.0 kg). Exclusion criteria included individuals with a history or presence of hypersensitivity to the study products or any related compound; allergic reaction to heparin; clinically significant abnormal laboratory values during a screening visit; any disease or drug therapy; alcohol or drugs addiction; difficulty in donating blood or having donated blood 90 days prior to the start of the study; participation in any other clinical study involving drug administration where blood samples were collected 90 days preceding the start of the study.

Mean demographic characteristics of the enrolled volunteers are reported in Table 1. The health condition of each volunteer was ascertained by subjective and objective examinations, including vital signs, blood and complete urine analysis. The same examinations were repeated at the end of the trial. Volunteers were adequately informed about potential benefits and adverse effects that might occur after ingesting the investigational products. 

### 4.3. Treatment

The investigational products (T and R) used for this trial were supplied by Giellepi S.p.A. Health Science (Lissone, Italy). Test product was a tablet containing µSmin^®^ Plus, a flavonoids complex standardized in diosmin (500 mg of micronized diosmin per tablet; particle size 95% lower than 5 µm), granulated (using aqueous solution of maltodextrin as a binder) and with calcium carbonate as buffering agent. Reference product was an identical tablet (same shape, dimension and color) containing the same amount of diosmin (500 mg) as unformulated micronized diosmin (particle size 95% lower than 5 µm). 

During each treatment period, all volunteers received a single oral administration of either T or R during the fasted state, according to a computer-generated randomization list (SAS^®^ software, version 9.4, SAS Institute Inc., Cary, NC, USA). Investigators were blinded during the entire trial process.

### 4.5. Blood Collection 

In each period, one blood sample (6 mL) was collected immediately before product administration (baseline). The other blood samples were collected at 0.25, 0.50, 0.75, 1.00, 1.25, 1.50, 1.75, 2.00, 3.00, 4.00, 6.00, 8.00, 10.00, 12.00, 16.00, 24.00, 30.00, 36.00, 48.00, 60.00, 72.00, 96.00 and 120.00 h post-dosing, into K_2_EDTA vacutainers, through an indwelling catheter placed in the subject’s forearm vein during the first 24 h. During this period, subjects were housed in a clinical facility, consumed standard meals and water was ad libitum. The remaining samples (from 30.00 to 120.00 h were collected by direct venipuncture and subjects returned to the clinical facility for sampling. Blood samples were immediately centrifuged at 4000 rpm for 10 min at 4 °C. Plasma was separated, collected in tubes and stored at −80 °C until chemical analysis.

### 4.6. Determination of Diosmetin in Plasma Samples

Enzymatic incubation was followed by a simple liquid-liquid extraction process to extract diosmetin from plasma samples. Diosmin is not absorbed along the gastro-intestinal tract and only its aglycone (diosmetin) is able to pass through the intestinal barrier and, like other flavonoids, circulates in the blood flow as glucuronide conjugates. Therefore, enzymatic pre-treatment of plasma samples is needed in order to liberate diosmetin (deconjugation process) and detect its plasmatic concentration. To this end, 50 µL of β-glucuronidase (1000 IU) were added to 100 µL of plasma and vortex-mixed. Then, ammonium formate 0.1 M (20 µL) was added and the mixture incubated for 2 h at 55 °C. After incubation, ammonium hydroxide 0.1 M (75 µL) and deuterated diosmetin (50 µL; 150 mg/mL) as internal standard (IS) were added for 2 min. Thereafter, isopropanol (2 mL) was added and the mixture centrifuged for 10 min at 4 °C. The supernatant aliquot (1.8 mL) was evaporated to dryness at 40 °C and reconstituted with mobile phase (200 µL). An aliquot (10 µL) was transferred into sampler vials for LC-MS/MS analysis. The chemical analysis was carried out using a LC-MS/MS system (LCMS-8040, Shimadzu, Kyoto, Japan) with Kinetex F5 (Phenomenex) column (3.0 μm and 50 × 4.6 mm). Isocratic elution was carried out using acetonitrile (5 mM) and ammonium acetate (80:20, *v*/*v*) at a flow rate of 0.5 mL/min. The analytes were detected using electrospray ionization in positive ion multiple rotation monitoring (MRM) mode.

The analytical method for the analysis of plasma samples was tested for linearity, precision, accuracy, recovery, lower limit of quantification and lower limit of detection. A calibration curve was constructed and quality control samples spiked directly with diosmetin, using the concentrations stated above, without the deconjugation process.

### 4.7. Pharmacokinetic Analysis

The maximum (or peak) plasma concentration (C_max_) and the time to reach it (t_max_), the area under the plasma concentration-time curve (AUC_0-t_) from time zero to the time of the last detected plasma concentration, and the apparent elimination rate constant (k_el_) were calculated with WinNonlin software using non-compartmental analysis. K_el_ was used to calculate the half-life (t_1/2_) as 0.693/k_el_. The extent of relative oral bioavailability (F) was calculated by dividing AUCs obtained for the test and reference products.

### 4.8. Safety Evaluation

Potential side effects were monitored during the entire course of the study. Adverse effects were considered severe if fatal, life threatening, requiring hospitalization, leading to permanent handicaps or congenital abnormalities.

### 4.9. Statistical Analysis

Statistical analysis was carried out using the SAS^®^ statistical software, version 9.4 (SAS Institute Inc.). The descriptive statistics (mean, median, minimum, maximum, standard deviation and coefficient of variation) for the pharmacokinetic parameters (primary parameters: C_max_, AUC_0-t_, and secondary parameters: t_max_, t_1/2_, and K_el_) were calculated for both tested formulation and reference. The Ln-transformed pharmacokinetic parameters (C_max_ and AUC_0-t_) were analyzed using an ANOVA model. To test the two, one-sided tests for ratio analysis, 90% confidence intervals for the difference between treatment’s least-square mean was calculated for Ln-transformed C_max_, AUC_0-t_. The confidence interval is expressed as a percentage difference.

## Figures and Tables

**Table 1:** Demographic data of subjects (*n* = 16) enrolled in the clinical trial. Data are shown as mean values ± standard deviations (SD). Coefficient of variation (CV%), minimum and maximum values are also shown.

	Age (Years)	Height (cm)	Weight (kg)	BMI (kg/m^2^)
Mean	29.94	166.00	63.69	23.04
SD	6.12	6.00	6.96	1.78
Min	19	156.00	51.00	19.69
Max	41	180.00	79.00	24.69
CV (%)	20.43	3.55	10.92	7.70

**Table 2:** Linearity, precision and accuracy for detection of diosmetin in plasma samples.

Theoretical Concentrations (ng/mL)	Mean Concentrations (ng/mL)	SD	Precision (%)	Accuracy (%)
0.506	0.513	0.016	3.100	101.320
1.011	0.984	0.051	5.210	97.300
3.678	3.637	0.128	3.530	98.890
9.195	9.398	1.033	10.990	102.210
26.271	27.595	0.235	0.850	105.040
65.677	68.915	5.001	7.260	104.930
164.193	169.634	9.397	5.540	103.310
211.863	184.436	4.855	2.630	87.050

**Table 3:** Pharmacokinetic parameters of diosmetin after oral administration of micronized diosmin (T and R).

PK Parameter	Test		Reference	
	Mean ± SD	CV (%)	Mean ± SD	CV (%)
C_max_ (ng/mL)	50.3 ± 22.6	88.3	2.4 ± 1.9	195.8
AUC_0-t_ (ng·mL^−1^·h)	298.4 ± 163.7	81.0	31.9 ± 100.4	314.7
t_max_ (h)	2.2 ± 2.9	131.8	nc *	-

* nc: not calculated.

## Abbreviations

AUC	area under the plasma concentration-time curve
BMI	body mass index
C_max_	maximum plasma concentration
CV%	coefficient of variation
CVI	Chronic venous insufficiency
F	bioavailability
HPLC-MS	high performance liquid chromatography-mass spectrometry
IS	internal standard
IU	international units
Kel	elimination constant
LC-MS/MS	liquid chromatography-tandem mass spectrometry
LOQ	limit of quantification
MRM	multiple rotation monitoring
nc	not calculable
PAMPA	parallel artificial membrane permeability assay
pK_a_	ionization constant
R	reference product
SD	standard deviation
T	tested formulation
t_max_	time needed to reach the C_max_
t_1/2_	half-life

## References

[1] Caristi C., Bellocco E., Panzera V., Toscano G., Vadalà R., Leuzzi U. (2003). Flavonoids detection by HPLC-DAD-MS-MS in lemon juices from Sicilian cultivars. J. Agric. Food Chem..

[2] Kanaze F.I., Gabrieli C., Kokkalou E., Georgarakis M., Niopas I. (2003). Simultaneous reversed-phase high-performance liquid chromatographic method for the determination of diosmin, hesperidin and naringin in different citrus fruit juices and pharmaceutical formulations. J. Pharm. Biomed. Anal..

[3] Marín F.R., Del Río J.A. (2001). Selection of hybrids and edible citrus species with a high content in the diosmin functional compound. Modulating effect of plant growth regulators on contents. J. Agric. Food Chem..

[4] Bush R., Comerota A., Meissner M., Raffetto J.D., Hahn S.R., Freeman K. (2017). Recommendations for the medical management of chronic venous disease: The role of Micronized Purified Flavanoid Fraction (MPFF). Phlebology.

[5] Garner R.C., Garner J.V., Gregory S., Whattam M., Calam A., Leong D. (2002). Comparison of the absorption of micronized (Daflon 500 mg) and nonmicronized ^14^C-diosmin tablets after oral administration to healthy volunteers by accelerator mass spectrometry and liquid scintillation counting. J. Pharm. Sci..

[6] Patel K., Gadewar M., Tahilyani V., Patel D.K. (2013). A review on pharmacological and analytical aspects of diosmetin: A concise report. Chin. J. Integr. Med..

[7] Silvestro L., Tarcomnicu I., Dulea C., Attili N.R., Ciuca V., Peru D., Rizea Savu S. (2013). Confirmation of diosmetin 3-*O*-glucuronide as major metabolite of diosmin in humans, using micro-liquid-chromatography-mass spectrometry and ion mobility mass spectrometry. Anal. Bioanal. Chem..

[8] Koutsos A., Tuohy K.M., Lovegrove J.A. (2015). Apples and cardiovascular health-is the gut microbiota a core consideration?. Nutrients.

[9] Muller M., Zartl B., Schleritzko A., Stenzl M., Viernstein H., Unger F.M. (2018). Rhamnosidase activity of selected probiotics and their ability to hydrolyse flavonoid rhamnoglucosides. Bioprocess Biosyst. Eng..

[10] Yang J., Qian D., Jiang S., Shang E.X., Guo J., Duan J.A. (2012). Identification of rutin deglycosylated metabolites produced by human intestinal bacteria using UPLC-Q-TOF/MS. J. Chromatogr. B.

[11] Zhang R., Zhang B.L., Xie T., Li G.C., Tuo Y., Xiang Y.T. (2015). Biotransformation of rutin to isoquercitrin using recombinant α-L-rhamnosidase from *Bifidobacterium breve*. Biotechnol. Lett..

[12] Bredsdorff L., Nielsen I.L., Rasmussen S.E., Cornett C., Barron D., Bouisset F., Offord E., Williamson G. (2010). Absorption, conjugation and excretion of the flavanones, naringenin and hesperetin from alpha-rhamnosidase-treated orange juice in human subjects. Br. J. Nutr..

[13] Monti D., Pisvejcová A., Kren V., Lama M., Riva S. (2004). Generation of an alpha-L-rhamnosidase library and its application for the selective derhamnosylation of natural products. Biotechnol. Bioeng..

[14] Zverlov V.V., Hertel C., Bronnenmeier K., Hroch A., Kellermann J., Schwarz W.H. (2000). The thermostable alpha-L-rhamnosidase RamA of *Clostridium stercorarium*: Biochemical characterization and primary structure of a bacterial alpha-L-rhamnoside hydrolase, a new type of inverting glycoside hydrolase. Mol. Microbiol..

[15] Serra H., Mendes T., Bronze M.R., Simplício A.L. (2008). Prediction of intestinal absorption and metabolism of pharmacologically active flavones and flavanones. Bioorg. Med. Chem..

[16] Freag M.S., Elnaggar Y.S., Abdallah O.Y. (2013). Development of novel polymer-stabilized diosmin nanosuspensions: In vitro appraisal and ex vivo permeation. Int. J. Pharm..

[17] Takahashi A.I., Rebello F.L., Dutra Duque M., Consiglieri V.O., Gomez Ferraz H. (2012). Using fluid bed granulation to improve the dissolution of poorly water-soluble drugs. Braz. Arch. Biol. Technol..

[18] Russo R., Mancinelli A., Ciccone M., Terruzzi F., Pisano C., Severino L. (2015). Pharmacokinetic profile of µSMIN Plus™, a new micronized diosmin formulation, after oral administration in rats. Nat. Prod. Commun..

